# Antimicrobial use in acute care hospitals: national point prevalence survey on healthcare-associated infections and antimicrobial use, Switzerland, 2017

**DOI:** 10.2807/1560-7917.ES.2019.24.33.1900015

**Published:** 2019-08-15

**Authors:** Walter Zingg, Aliki Metsini, Céline Gardiol, Carlo Balmelli, Michael Behnke, Nicolas Troillet, Andreas Widmer, Didier Pittet, on behalf of the Swissnoso Network

**Affiliations:** 1 Infection Control Programme and WHO Collaborating Centre on Patient Safety, University of Geneva Hospitals, Geneva, Switzerland; 2 Imperial College London, London, United Kingdom; 3 Swiss Federal Office of Public Health, Bern, Switzerland; 4 Infection Control Programme, Cantonal Hospital Authority, Ticino, Switzerland; 5 Institute of Hygiene and Environmental Medicine, Charité University Medicine Berlin, Berlin, Germany; 6 Department of Infectious Diseases, Central Institute, Valais Hospital, Sion, Switzerland; 7 Division of Infectious Diseases and Hospital Epidemiology, University Hospital Basel, Basel, Switzerland; 8 Members of the network are acknowledged at the end of the article

**Keywords:** point prevalence survey, Switzerland, antimicrobial consumption, antimicrobial resistance, antimicrobial stewardship, acute care, Swissnoso, ECDC

## Abstract

**Background:**

A point prevalence survey (PPS) on healthcare-associated infections (HAI) and antimicrobial use was conducted in Swiss acute care hospitals in 2017.

**Aim:**

Our objective was to assess antimicrobial use in Swiss acute care hospitals.

**Methods:**

All patients hospitalised in any acute care hospital in Switzerland were eligible. We used the most recent version of the PPS protocol of the European Centre for Disease Prevention and Control.

**Results:**

Data from 12,931 patients of 96 hospitals were collected. Of these, 4,265 (33%; 95% confidence interval (CI): 32.2–33.8) were on 5,354 antimicrobials for 4,487 indications. Most of the 2,808 therapeutic indications addressed 1,886 community-acquired infections (67.2%; 95% CI: 65.4–68.9). Of the 1,176 surgical prophylaxes, 350 (29.8%; 95% CI: 27.1–32.4) exceeded the duration of 1 day. Of the 1,090 antimicrobial regimens that were changed, 309 (28.3%; 95% CI: 25.7–31.0) were escalated and 337 (30.9%; 95% CI: 28.2–33.7) were de-escalated. Amoxicillin/clavulanic acid was the most frequent antimicrobial (18.8%; 95% CI: 17.7–19.8), prescribed mainly for therapeutic indications (76.0%; 95% CI: 73.3–78.7). A total of 1,931 (37.4%; 95% CI: 36.1–38.8) of the 5,158 antimicrobials for systemic use were broad-spectrum antibiotics, most frequently third- and fourth-generation cephalosporins (35.9%; 95% CI: 33.8–38.1).

**Conclusions:**

Antimicrobial consumption was at European average, the use of broad-spectrum antibiotics in the lower third. Swiss acute care hospitals should invest in antimicrobial stewardship, particularly in reducing the use of broad-spectrum antibiotics.

## Introduction

In the European Union (EU), one or more antimicrobials are given to at least a third of inpatients on any hospital day [1], which makes them one of the most frequently prescribed drug classes in acute care hospitals. Antimicrobial resistance (AMR) has become a global problem, and knowledge about the local and regional situation is important to guide therapeutic decisions [2]. Antimicrobials, particularly broad-spectrum antibiotics, drive the emergence of AMR, mostly through selection pressure [3,4], and favour infections caused by *Clostridium difficile* [5-7] or fungi [8]. Infections that are due to Gram-positive or Gram-negative multidrug-resistant microorganisms (MDRO) prolong hospital stay and increase mortality [9-11]. Antimicrobial stewardship programmes, if resulting in measurable reduction and judicious use of antimicrobials, effectively reduce AMR [12].

For many years, point prevalence surveys (PPS) have been conducted for the surveillance of antimicrobial use, first in outpatient care [13], later in hospitals [14]. The European Surveillance of Antimicrobial Consumption Network (ESAC-Net) monitors the use of antimicrobials in the European Union (EU) and the European Economic Area (EEA), but does not provide clinical data to assess the appropriateness of antimicrobial prescriptions [15,16]. This gap was filled by combining prevalence surveys on healthcare-associated infections (HAI) and on antimicrobial use. In 2011 and 2012, the EU Member States, Iceland, Norway, and Croatia participated in such a combined PPS, the first PPS by the European Centre for Disease Prevention and Control (ECDC PPS) on HAI and antimicrobial use [1,17]. The HAI prevalence at that time was 6.0%, and 35.0% of patients received one or more antimicrobials [1]. Five years later, the second ECDC PPS was conducted in the EU and EEA countries and in some EU candidate countries [16,18,19]. The HAI prevalence was 6.5%, and 30.3% of patients received one or more antimicrobials. Switzerland was part neither of the first nor the second ECDC PPS.

In January 2013, the Swiss Federal Council passed its ‘Health 2020’ agenda to set priorities in healthcare management in Switzerland [20]. The Federal Office of Public Health (FOPH), together with various stakeholders from health delivery in Switzerland, defined two strategies for HAI prevention and AMR: the ‘Strategy NOSO’ and the ‘Strategy StAR’ [21,22]. Those national strategies aim at reducing HAIs and containing the emergence and spread of AMR in the various healthcare settings in Switzerland. FOPH mandated Swissnoso, a non-profit association of leading experts in the field of infection prevention and control and infectious diseases in Switzerland (www.swissnoso.ch), to perform a national PPS (CH-PPS) on HAI and the use of antimicrobials, simultaneously with the second ECDC PPS and using the same protocol. The objective was to assess the situation of HAI and antimicrobial use in Swiss acute care hospitals [18]. 

## Methods

### Setting and study population

In 2016, a pilot survey was performed in three large acute care hospitals in Switzerland with the objective to test the ECDC PPS protocol [23]. The ECDC protocol version 5.3 was translated into French, German and Italian (www.swissnoso.ch) [18]. No methodological changes were made in the CH-PPS protocol. In December 2016, all 187 acute care hospitals in Switzerland were invited to participate in the CH-PPS, planned for the second trimester of 2017. Participation was voluntary, and hospitals were compensated for their efforts: CHF 200 (EUR 176) as a minimum fee plus CHF 5 (EUR 4.40) per additional included patient. 

## Data collection 

The CH-PPS coordination centre was established for project management, education and training, data management, and data analysis. The centre organised seven interactive training workshops for hospital investigators and data collectors: four in the German-speaking region, two in the French-speaking region, and one in the Italian-speaking region of Switzerland. The courses used a structured methodology, encouraging a participative, problem-solving approach by discussing clinical cases and using the database interactively. The main data collection started on 1 April 2017 and ended on 30 June 2017, either using the specific CH-PPS case report forms followed by data entry to the electronic CH-PPS database or by direct data entry. The database featured plausibility algorithms and was provided by the Institute of Hygiene and Environmental Medicine at Charité Universitätsmedizin Berlin, Berlin, Germany [24]. During the data entry period, hospitals had the option to download their own data in different formats (HTML, CSV, pdf).

All patients hospitalised in acute care were eligible for inclusion if admitted to the ward before or at 08:00 and if not discharged during the survey day. Patients in the emergency room, in psychiatry and in outpatient care were excluded. Patients in rehabilitation and long-term care were included if such specialties were part of the acute care hospital. Patient information was collected using an individual patient form on demographic data, HAI, medical device and antimicrobial use, and data on microorganisms with information on AMR. The detailed methodology is described elsewhere [23]. In addition, the hospitals provided data on antimicrobial policy, antimicrobial stewardship and additional activities in the context of antimicrobial use.

For antimicrobial use, the following data were collected: agent, route, dosage and indication as judged by the prescriber (treatment of community-, hospital- or long-term care-acquired infection, surgical or medical prophylaxis), diagnosis by anatomical site in case of treatment, documentation of the reason for antimicrobial prescription in the patient chart and change of the current antimicrobial regime. In case of changed regime, additional information on the last change was obtained: escalation, de-escalation, change from intravenous to oral, or any other type of change. The prevalence of antimicrobial use was reported as the percentage of patients receiving one or more antimicrobials on the survey day. The Anatomical Therapeutic Chemical (ATC) classification system was used for data analysis. Drugs were defined to the 5th level of the ATC classification. The relative frequencies of individual antimicrobials representing the 75% most commonly used drugs (DU75%) were calculated [25]. Defined daily doses (DDD) per 100 adult (≥ 18 years) patient-days were calculated using the 2018 ATC/DDD classification issued by the World Health Organization Collaborating Centre for Drug Statistics and Methodology [26]. Outcomes were stratified by hospital size (small: < 200 beds; medium size: 200–650 beds; large: > 650 beds) and hospital type (primary care, secondary care, tertiary care, specialised care). We also calculated the proportion of broad-spectrum antibiotics among all antibiotics for systemic use (ATC J01). The following antimicrobial groups and agents were considered broad-spectrum antibiotics: piperacillin/tazobactam, third- and fourth-generation cephalosporins, monobactams, carbapenems, fluoroquinolones, glycopeptides, polymyxins, daptomycin and oxazolidinones [27].

### Data analysis

Descriptive data are reported as medians with interquartile range (IQR) or means with 95% confidence intervals (CI) where appropriate. Statistical analysis of patient characteristics, frequencies of indications and therapeutic diagnoses, and frequencies of broad-spectrum antibiotic use relative to hospital size and types was done by using the non-parametric Kruskal–Wallis test. A rapidly or ultimately fatal McCabe score (expected fatal outcome within 1 or 5 years, respectively) [28], age groups (0–17, 18–40, 41–60, 61–80, > 80 years), hospitalisation in an intensive care unit (ICU) on the survey day, exposure to a medical device (peripheral or central venous catheter, urinary catheter, endotracheal tube) on the survey day, having undergone National Healthcare Safety Network (NHSN) surgery [29] since hospital admission and private-for-profit ownership of the hospital were tested in a univariable logistic regression analysis as risk factors for both receiving one or more antimicrobials and receiving systemic broad-spectrum antibiotics. Variables with a significance level of p ≤ 0.2 were tested in a multivariable model. Observations were clustered at hospital level; a two-sided p value of 0.05 was considered significant. Data analysis was performed using STATA version 13 (STATA Corporation, College Station, United States).

### Ethical statement

No institutional review board approval was deemed necessary, similar to the ECDC PPS, and given the quality improvement character of the survey. Only anonymous patient and ward data were collected and analysed.

## Results

Ninety-six hospitals with 12,931 patients participated in the survey: 63 (65.6%) small hospitals with 3,516 (27.2%) patients, 26 (27.1%) medium-size hospitals with 4,380 (33.9%) patients, and 7 (7.3%) large hospitals with 5,035 (38.9%) patients [30]. Median age was 68 years (IQR: 48–80). Patient characteristics of the participants of the national PPS are published elsewhere [30].

Table 1 summarises antimicrobial use and hospital indicators on antimicrobial stewardship and surveillance, stratified by hospital size and hospital category. A total of 4,265 patients received one or more antimicrobials on the survey day, resulting in a prevalence of 33.0% (95% CI: 32.2–33.8). The prevalence of patients on one or more antimicrobials in small, medium size and large hospitals was 33.4% (n = 1,175; 95% CI: 31.9–35.0), 34.4% (n = 1,506; 95% CI: 33.0–35.8) and 31.5% (n = 1,584; 95% CI: 30.2–32.7), respectively (p = 0.009). Of the 358 paediatric patients, 69 (19.3%; 95% CI: 15.3–23.7) received one or more antimicrobials on the survey day, while this proportion was 33.4% (95% CI: 32.6–34.2) in adults. Overall DDD were 59.4 per 100 patients (7,089 DDD in 11,941 adults). Re-evaluation of antimicrobial treatment after 48–72 h was performed by nine of the 46 hospitals reporting on this indicator. Only six hospitals (two per hospital size category) had formal positions for antimicrobial stewardship. Guidelines on antimicrobial use were available in 70.5% of 95 hospitals reporting on this indicator, with higher proportions in large and tertiary care hospitals. Surveillance of AMR and antimicrobial use was reported by 57 (59.4%) and 43 (44.8%) hospitals, respectively; again more often in large and tertiary care hospitals.

**Table 1:** Antimicrobial use and hospital indicators on antimicrobial stewardship and surveillance, by hospital size and hospital category, national point prevalence survey on antimicrobial use, Switzerland, 2017 (n = 12,931 patients)

	Hospitals	Patients	Antimicrobial use				Antimicrobial stewardship			Surveillance^a^	
			Patients ≥ 1 AM			DDD^b^	AMS consultant	Post-prescription review^c^	AM guidelines	AM use	AMR
	n	n (adults^b^)	n	%	95% CI	n/100 PD	FTE/250 beds^d^	n/N	n/N	n/N	n/N
Hospital size											
< 200 beds	63	3,516 (3,155)	1,175	33.4	31.9–35.0	51.9	0.01	4/28	40/63	23/96	36/96
200–650 beds	26	4,380 (4,137)	1,506	34.4	33.0–35.8	57.7	0.03	5/16	21/26	13/96	15/96
> 650 beds	7	5,035 (4,649)	1,584	31.5	30.2–32.7	66.1	0.01	0/2	6/6	7/96	6/96
Hospital type											
Primary care	38	2,694 (2,510)	865	32.1	30.3–33.9	54.6	0.02	4/18	28/38	14/96	22/96
Secondary care	40	4,325 (4,085)	1,465	33.9	32.5–35.3	53.8	0.02	4/18	30/40	21/96	24/96
Tertiary care	11	5,549 (4,984)	1,810	32.6	31.4–33.9	67.6	0.01	0/4	8/10	8/96	9/96
Specialised care	7	363 (362)	125	34.4	29.5–39.3	45.0	0.00	1/6	1/7	0/96	2/96
**Total**	**96**	**12,931 (11,941)**	**4,265**	**33.0**	**32.2–33.8**	**59.4**	**0.02**	**9/46**	**67/95**	**43/96**	**57/96**

AM: antimicrobial; AMR: antimicrobial resistance; AMS: antimicrobial stewardship; CI: confidence interval; DDD: defined daily doses; FTE: full-time equivalent; PD: patient-days.

^a^ Participation in a surveillance network.

^b^ Patients ≥ 18 years.

^c^ Formal post-prescription review after 72–96 h in any setting (or hospital-wide); data provided by 46 hospitals only.

^d^ Only six hospitals had a formal AMS consultant.

The 4,265 patients on antimicrobials received 5,354 antimicrobial agents for 4,487 indications. Table 2 summarises indications for antimicrobial use and diagnoses for therapeutic indications. Numbers and proportions of indications for therapeutic use, prophylaxis and other or unknown indications were 2,808 (62.6%; 95% CI: 61.2–64.0), 1,533 (34.2%; 95% CI: 32.8–35.6) and 146 (3.3%; 95% CI: 2.7–3.8), respectively. Most of the 2,808 therapeutic indications were for community-acquired infections (n = 1,886; 67.2%; 95% CI: 65.4–68.9) (Supplementary Table 1) and predominantly for treating lower respiratory tract (n = 678; 24.1%; 95% CI: 22.6–25.8), urinary tract (n = 448; 16.0%; 95% CI: 14.6–17.4) and bloodstream infections (n = 286; 10.2%; 95% CI: 9.1–11.4) (Table 2). Most of the 1,533 prophylactic indications were for surgical procedures (n = 1,176; 76.7%; 95% CI: 74.6–78.8), of which 677 (57.6%; 95% CI: 54.7–60.4) were administered as single dose, 149 (12.7%; 95% CI: 10.8–14.6) during 1 day and 350 (29.8%; 95% CI: 27.1–32.4) for more than 1 day (Supplementary Table 1). While the proportion of prolonged surgical prophylaxis in small hospitals (87/437; 19.9%; 95% CI: 16.1–23.7) and medium-size hospitals (104/424; 24.5%; 95% CI: 20.4–28.6) was small, this proportion was statistically significantly higher in large hospitals (159/315; 50.5%; 95% CI: 44.9–56.0). A total of 1,542 (28.8%; 27.6–30.0) of the 5,354 antimicrobials were administered by the oral route, with the highest proportion in rehabilitation (59/84; 70.2%; 95% CI: 60.3–80.2), followed by geriatrics (70/133; 52.6%; 95% CI: 44.0–61.2) and internal medicine (814/2,066; 39.4%; 95% CI: 37.3–41.5).

**Table 2:** Indications for antimicrobial use and diagnoses for therapeutic treatment, by hospital size, national point prevalence survey on antimicrobial use, Switzerland, 2017 (n = 4,487)

	All hospitals			Hospital size									p value
				< 200 beds			200–650 beds			> 650 beds			
	n	%	95% CI	n	%	95% CI	n	%	95% CI	n	%	95% CI	
Indications	n = 4,487			n = 1,216			n = 1,545			n = 1,726			
Community-acquired infection	1,886	42.0	40.6–43.5	513	42.2	39.4–45.0	726	47.0	44.5–49.5	647	37.5	35.2–39.8	< 0.001
Healthcare-associated infection^a^	852	19.0	17.8–20.1	173	14.2	12.2–16.2	245	15.9	14.0–17.7	434	25.1	23.1–27.2	< 0.001
LTCF-acquired infection	70	1.6	1.2–1.9	25	2.1	1.3–2.9	24	1.6	0.9–2.2	21	1.2	0.7–1.7	0.280
Medical prophylaxis	357	8.0	7.2–8.7	44	3.6	2.6–4.7	74	4.8	3.7–5.9	239	13.8	12.2–15.5	< 0.001
Surgical prophylaxis	1,176	26.2	24.9–27.5	437	35.9	33.2–38.6	424	27.4	25.2–29.7	315	18.3	16.4–20.1	< 0.001
Other or unknown indication	146	3.3	2.7–3.8	24	2.0	1.2–2.8	52	3.4	2.5–4.3	70	4.1	3.1–5.0	0.007
Diagnoses^b^	n = 2,808			n = 711			n = 995			n = 1,102			
Bloodstream infection/sepsis	286	10.2	9.1–11.4	56	7.9	6.0–10.1	109	11.0	9.1–13.1	121	11.0	9.2–13.0	0.013
Lower respiratory tract infection	678	24.1	22.6–25.8	173	24.3	21.2–27.7	232	23.3	20.7–26.1	273	24.8	22.2–27.4	0.491
Urinary tract infection	448	16.0	14.6–17.4	151	21.3	18.3–24.4	164	16.5	14.2–18.9	133	12.1	10.2–14.1	< 0.001
Soft tissue infection	276	9.8	8.8–11.0	78	11.0	8.8–13.5	118	11.9	9.9–14.0	80	7.3	5.8–9.0	0.002
Bone and joint infection	140	5.0	4.2–5.9	48	6.8	5.0–8.9	33	3.3	2.3–4.6	59	5.4	4.1–6.9	0.017
Surgical site infection	250	8.9	7.9–10.0	52	7.3	5.5–9.5	90	9.0	7.3–11.0	108	9.8	8.1–11.7	0.061
Intra-abdominal infection	266	9.5	8.4–10.6	59	8.3	6.4–10.6	101	10.2	8.3–12.2	106	9.6	7.9–11.5	0.158
Gastrointestinal infection	116	4.1	3.4–4.9	28	3.9	2.6–5.6	38	3.8	2.7–5.2	50	4.5	3.4–5.9	0.565
Vascular infection	71	2.5	2.0–3.2	10	1.4	0.7–2.6	24	2.4	1.6–3.6	37	3.4	2.4–4.6	0.829
Other infection	277	9.9	8.8–11.0	56	7.9	6.0–10.1	86	8.6	7.010.6–	135	12.3	10.4–14.3	0.001

CI: confidence interval; LTCF: long-term care facility.

^a^ Healthcare-associated as documented in the patient chart.

^b^ Diagnoses: applicable to community-acquired infections, healthcare-associated infections, and LTCF-acquired infections (2,808 in total).

A quarter of antimicrobial regimens were changed (1,090/4,487; 24.3%; 95% CI: 23.0–25.5). Most often, change occurred in surgical site (129/250; 51.6%; 95% CI: 45.4–57.8), bloodstream (126/286; 44.1%; 95% CI: 38.3–49.8), intra-abdominal (108/266; 40.6%; 95% CI: 34.7–46.5) and healthcare-associated infections (342/852; 40.1%; 95% CI: 36.8–43.4) (Table 3). A total of 309 (28.3%; 95% CI: 25.7–31.0) and 337 (30.9%; 95% CI: 28.2–33.7) of the 1,090 changed antimicrobial regimens were escalated and de-escalated, respectively. Escalation was most often reported in intensive care (25/64; 39.1%; 95% CI: 26.8–51.3), healthcare-associated (136/342; 39.8%; 95% CI: 34.6–45.0) and intra-abdominal infections (40/108; 37.0%; 95% CI: 27.8–46.3) (Table 3). De-escalation was reported most often for bloodstream (54/126; 42.9; 95% CI: 34.1–51.6) and urinary tract infections (49/129; 38.0%; 95% CI: 29.5–46.5) (Table 3). Of the 3,479 antimicrobials prescribed for the 2,808 therapeutic indications, 255 (7.3%; 95% CI: 6.5–8.2) were switched from intravenous to oral and 40 (1.2%; 95% CI: 0.8–1.5) were changed because of side effects. The majority of 3,903 (87.0%; 95% CI: 86.0–88.0) of the 4,487 antimicrobial indications were documented in the patient charts, without differences across hospital size; however, specialised hospitals documented significantly more indications (128/135; 94.8%; 95% CI: 91.0–98.6) compared with other hospital types (Table 3).

**Table 3:** Change of antimicrobial treatment, national point prevalence survey on antimicrobial use, Switzerland, 2017 (n =  4,487)

	Indications	Reason in notes			Any change			Escalation			De-escalation		
	total	n	% of total	95% CI	n	% of total	95% CI	n	% of change	95% CI	n	% of change	95% CI
Hospital size													
< 200 beds	1,216	1,040	85.5	83.5–87.5	249	20.5	18.2–22.7	77	30.9	25.1–36.7	63	25.3	19.9–30.7
200–650 beds	1,545	1,355	87.7	86.1–89.3	381	24.7	22.5–26.8	87	22.8	18.6–27.1	126	33.1	28.3–37.8
> 650 beds	1,726	1,508	87.4	85.8–88.9	460	26.7	24.6–28.7	142	30.9	26.6–35.1	155	33.7	29.4–38.0
Hospital type													
Primary care	886	759	85.7	83.4–88.0	188	21.2	18.5–23.9	58	30.9	24.2–37.5	46	24.5	18.3–30.7
Secondary care	1,510	1,323	87.6	86.0–89.3	369	24.4	22.3–26.6	86	23.3	19.0–27.6	127	34.4	29.5–39.3
Tertiary care	1,956	1,693	86.6	85.0–88.1	511	26.1	24.2–28.1	159	31.1	27.1–35.1	169	33.1	29.0–37.2
Specialised care	135	128	94.8	91.0–98.6	22	16.3	10.0–22.6	3	13.6	0.0–29.2	2	9.1	0.0–22.1
Indication/diagnosis													
Therapy	2,808	2,638	94.0	93.1–94.8	1,023	36.4	34.7–38.2	306	29.9	27.1–32.7	344	33.6	30.7–36.5
Community infection	1,886	1,781	94.4	93.4–95.5	652	34.6	32.4–36.7	163	25.0	21.7–28.3	235	36.0	32.3–37.7
Healthcare-associated infection	852	795	93.3	91.6–95.0	342	40.1	36.8–43.4	136	39.8	34.6–45.0	101	29.5	24.7–34.4
Bloodstream infection/sepsis	286	268	93.7	90.9–96.5	126	44.1	38.3–49.8	31	24.6	17.0–32.2	54	42.9	34.1–51.6
Lower respiratory tract infection	678	645	95.1	93.5–96.8	263	38.8	35.1–42.5	80	30.4	24.8–36.0	73	27.8	22.3–33.2
Urinary tract infection	448	423	94.4	92.3–96.6	129	28.8	24.6–33.0	38	29.5	21.5–37.4	49	38.0	29.5–46.5
Intra-abdominal infection	266	244	91.7	88.4–95.1	108	40.6	34.7–46.5	40	37.0	27.8–46.3	35	32.4	23.4–41.4
Surgical site infection	250	233	93.2	90.1–96.3	129	51.6	45.4–57.8	43	33.3	25.1–41.6	35	27.1	19.4–34.9
Patient specialty													
Intensive care	204	179	87.8	83.2–92.3	64	31.4	25.0–37.8	25	39.1	26.8–51.3	24	37.5	25.3–49.7
Surgery	2,133	1,789	83.9	82.3–85.4	418	19.6	17.9–21.3	118	28.2	23.9–32.6	121	29.0	24.6–33.3
Internal medicine	1,680	1,542	91.8	90.5–93.1	528	31.4	29.2–33.7	148	28.0	24.2–31.9	175	33.1	29.1–37.2
Paediatrics	69	55	79.7	70.0–89.4	9	13.0	4.9–21.2	2	22.2	0.0–56.1	2	22.2	0.0–56.1
Geriatrics	115	11	9.6	4.9–16.5	31	27.0	18.7–35.2	6	19.4	4.6–34.1	11	35.5	17.6–53.3
Rehabilitation	71	59	83.1	74.2–92.0	20	28.2	17.4–38.9	2	10.0	0.0–24.4	6	30.0	8.0–52.0
**Total**	**4,487**	**3,903**	**87.0**	**86.0–88.9**	**1,090**	**24.3**	**23.0–25.5**	**309**	**28.3**	**25.7–31.0**	337	**30.9**	**28.2–33.7**

**Figure 1:**
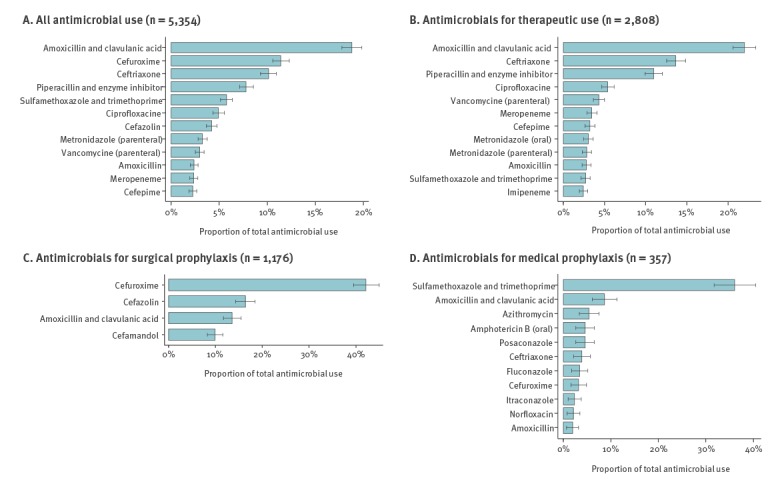
Antimicrobials accounting for 75% of antimicrobial use (DU 75%), national point prevalence survey on antimicrobial use, Switzerland, 2017 (n =  5,354)

Figure 1 summarises the DU75%, stratified according to therapeutic use, surgical prophylaxis and medical prophylaxis. Amoxicillin/clavulanic acid was the most common antimicrobial drug (1,006/5,354; 18.8%; 95% CI: 17.7–19.8), used mainly for therapeutic indications (765/1,006; 76.0%; 95% CI: 73.3–78.7) such as community-acquired lower respiratory tract infection (235/765; 30.7%; 95% CI: 27.5–34.1), followed by cefuroxime (612/5,354; 11.4%; 95% CI: 10.6–12.3), used mainly for surgical prophylaxis (528/612; 86.3%; 95% CI: 83.3–88.9), followed by ceftriaxone (542/5,354; 10.1%; 95% CI: 9.3–10.9), used mainly for therapeutic indications (476/542; 87.8%; 95% CI: 84.8–90.5) such as community-acquired urinary (145/476; 30.5%; 95% CI: 26.4–34.8) and lower respiratory tract infection (105/476; 22.1%; 95% CI: 18.4–26.1) (Supplementary Table 2). Combination of two or more than two antimicrobial agents was reported for, respectively, 638 (14.2%; 95% CI: 13.2–15.2) and 100 (2.2%; 95% CI: 1.8–2.7) of the 4,487 indications. The details of combined use of antimicrobial agents are summarised in Supplementary Table 3.

Bars and whiskers indicate antimicrobial use with 95% confidence intervals.

**Figure 2:**
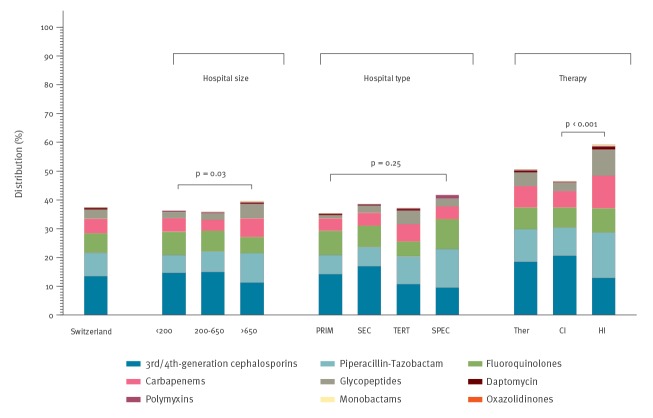
Proportion of broad-spectrum antibiotics among all antimicrobials for systemic use, national point prevalence survey on antimicrobial use, Switzerland, 2017 (n =  5,158)

CI: community infection; HI: healthcare-associated infection; SEC: secondary care; SPEC: specialty care; TERT: tertiary care; Ther: therapeutic use.

A total of 5,158 of the 5,354 antimicrobials were for systemic use (96.3%; 95% CI: 95.8–96.8). Figure 2 summarises the proportions of broad-spectrum antibiotics among all antimicrobials for systemic use, stratified by hospital size and category as well as therapeutic use for community- and healthcare-acquired infections. A total of 1,931 (37.4%; 95% CI: 36.1–38.8) antimicrobials were broad-spectrum antibiotics, most commonly third- and fourth-generation cephalosporins (694/1,931; 35.9%; 95% CI: 33.8–38.1), followed by piperacillin/tazobactam (419/1,931; 21.7%; 95% CI: 19.9–23.6) and quinolones (347/1,931; 18.0%; 95% CI: 16.3–19.8). Significantly more broad-spectrum antibiotics were used to treat HAI (606/1,025; 59.1%; 95% CI: 56.1–62.1) than to treat community-acquired infections (1,043/2,337; 44.6%; 95% CI: 44.6–48.7) (Figure 2).

Table 4 summarizes uni- and multivariable analyses to predict antmimicrobial use. Male sex, exposure to one or more medical devices, undergoing NHSN-surgery and hospitalisation in intensive care were independently associated with receiving one or more antimicrobials (Table 4). Older age, exposure to one or more medical devices, rapidly or ultimately fatal McCabe scores and admission to intensive care were independently associated with receiving broad-spectrum antibiotics (Table 4).

**Table 4:** Predictive variables for the use of one or more antimicrobials and for broad-spectrum antibiotics, national point prevalence survey on antimicrobial use, Switzerland, 2017 (n = 12,931)

Variable	Univariable analysis			Multivariable analysis		
	OR	95% CI	p value	OR	95% CI	p value
One or more antimicrobials						
Age^a^	1.07	1.00–1.13	0.037			
Male sex	1.53	1.40–1.66	< 0.001	1.37	1.25–1.49	< 0.001
Fatal McCabe score^b^	1.24	1.07–1.45	< 0.001			
Medical device^c^	6.75	6.00–7.65	< 0.001	6.21	5.49–7.02	< 0.001
NHSN surgery since admission	1.78	1.56–2.03	< 0.001	1.63	1.44–1.84	< 0.001
Hospitalisation in intensive care	3.48	2.74–4.42	< 0.001	1.77	1.44–2.18	< 0.001
Large hospital (> 650 beds)	0.89	0.65–1.23	0.487			
Tertiary care hospital	0.9)	0.72–1.31	0.851			
Private-for-profit ownership	0.89	0.66–1.20	0.437			
Broad-spectrum antibiotics						
Age^a^	1.22	1.13–1.33	< 0.001	1.17	1.09–1.26	< 0.001
Male sex	1.58	1.43–1.74	< 0.001	1.33	1.21–1.46	< 0.001
Fatal McCabe score^b^	1.69	1.43–1.99	< 0.001	1.36	1.16–1.60	< 0.001
Medical device^c^	10.64	8.49–13.3	< 0.001	9.35	7.43–11.78	< 0.001
NHSN surgery since admission	0.98	0.83–1.15	0.795			
Hospitalisation in intensive care	4.26	3.53–5.15	< 0.001	2.50	2.10–2.99	< 0.001
Large hospital (> 650 beds)	1.08	0.84–1.40	0.542			
Tertiary care hospital	1.00	0.78–1.28	0.997			
Private-for-profit ownership	0.92	0.70–1.22	0.562			

CI: confidence interval; NHSN: National Healthcare Safety Network; OR: odds ratio.

^a^ Age groups: 0–17, 18–40, 41–60, 61–80, > 80 years.

^b^ Rapidly (within 12 months) or ultimately (within 5 years) fatal disease.

^c^ Peripheral venous catheter, central venous catheter, urinary catheter or endotracheal tube.

### Benchmarking to the second ECDC point prevalence survey

**Figure 3:**
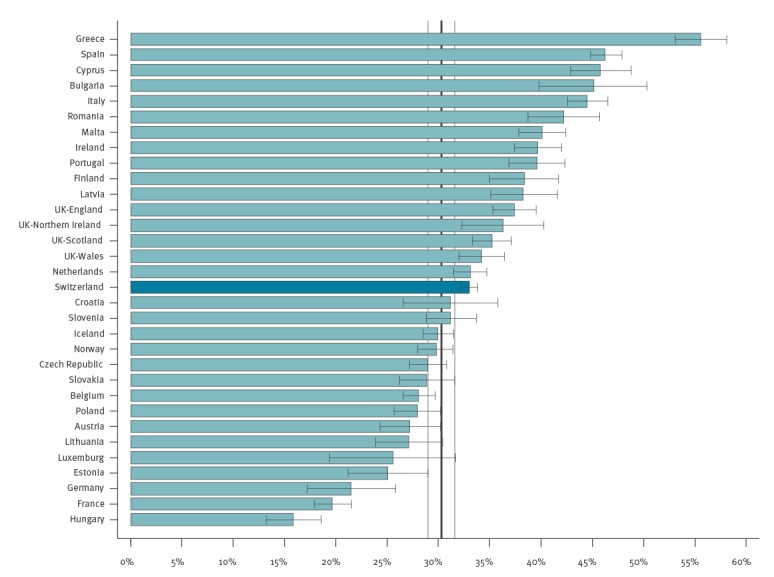
Prevalence of antimicrobial use in the Swiss and the ECDC point prevalence surveys combined

CI: confidence interval; ECDC: European Centre for Disease Prevention and Control; PPS: point prevalence survey.

Switzerland used the ECDC protocol and conducted the CH-PPS at the same time as the second ECDC PPS. Thus, data could be benchmarked to the EU and EEA countries. Figure 3 shows the position of Switzerland compared with the countries participating in the second ECDC PPS [16]. Antimicrobial use in Swiss acute care hospitals was at EU average (30.3%).

The prevalence indicates the proportion of patients receiving one or more antimicrobials at time of survey.

Bars and whiskers: prevalences with 95% CI by country. Vertical lines: mean prevalence and 95% CI across the countries participating in the 2016 and 2017 ECDC PPS. Source: [16].

## Discussion

This was the first national PPS on antimicrobial use in Swiss acute care hospitals. The data are representative for Switzerland, considering that almost 80% of the acute care hospitals with 100 beds or more contributed data to the survey. One in three patients in acute care hospitals received one or more antimicrobials, of which nearly 40% were broad-spectrum antibiotics. The most important indication for antimicrobial use was the treatment of community-acquired infections, mainly lower respiratory and urinary tract infections. One in five patients received antimicrobials for the treatment of an HAI, mostly broad-spectrum antibiotics. Surgical prophylaxis was the second most common indication for the use of antimicrobials, of which a third was prescribed for more than 1 day.

Antimicrobial consumption is also monitored by anresis, a comprehensive and representative surveillance system for antibiotic consumption and resistance in Switzerland (www.anresis.ch) collecting data from hospital pharmacies. The quantities of antimicrobials in grams are converted to DDD and expressed as DDD per 100 bed-days [31]. In 2017, 62 DDD per 100 bed-days were reported, which is very close to the 59 DDDs per 100 patient-days measured in our survey [32]. Anresis covers surveillance of AMR for most of the Swiss acute care hospitals, while surveillance of antimicrobial consumption is done by a voluntary sentinel network of 70 hospital pharmacies. Anresis does not provide clinical data. Furthermore, although the system observes both consumption and resistance, no patient-based link is provided to put antimicrobial use into clinical perspective. Compared with Europe, Switzerland had low formal staffing for antimicrobial stewardship (0.02/250 beds vs 0.37/250 beds), and a low proportion of hospitals with established formal post-prescription review (20% vs 52%).

Although antimicrobials were prescribed predominantly for therapeutic purposes both in Switzerland and in Europe, there are differences. The proportion of therapeutic indications in Switzerland (63%) was lower compared with Europe (71%), while the proportion of prophylaxis, particularly for surgery, was higher (34% vs 25%, respectively) [19]. A third of surgical prophylaxis was administered for more than 1 day, particularly in large and tertiary care hospitals, where such use contributed to more than half of surgical prophylaxis. The overall proportion of prolonged surgical prophylaxis, however, was lower than the European average (54%) and in Germany (54%), but similar to Poland and Wales [16,24]. However, Belgium, the Netherlands, Northern Ireland, Norway and Scotland had lower proportions. By applying best practice recommendations, the contribution of prolonged regimens to surgical prophylaxis could be reduced [33,34].

Amoxicillin/clavulanic acid was the most commonly prescribed antimicrobial not only in Switzerland but also in the EU/EEA, although the contribution of this agent to the total of antimicrobials was much higher in Switzerland (19% vs 11%) [16]. Cefuroxime, ceftriaxone and piperacillin/tazobactam were among the most frequently prescribed antimicrobials in both Switzerland and the EU/EEA, although the proportion of ceftriaxone in Switzerland was higher (11% vs 7%). The proportion of broad-spectrum antibiotics in Switzerland was lower than the European average (37% and 42%, respectively) [16] and in between its neighbouring countries Austria (35%) and Germany (47%). There was much variation for drug-combinations; the four most frequent combinations of two agents included metronidazole in combination with a third- or fourth-generation cephalosporin or a fluoroquinolone for the treatment of community-acquired intra-abdominal infections or surgical prophylaxis.

Our survey has limitations. Firstly, prevalence is a point estimate, which limits its generalisability. However, compared with PPS on HAI, a rare outcome, antimicrobial use is a frequent outcome and thus, variation is much lower. Secondly, variation in the application of the definitions on antimicrobial change must be assumed, as challenges with the CH-PPS protocol reported to the coordination centre focused often on interpreting escalation and de-escalation of antimicrobials.

From an organisational point of view, the survey was successful in achieving the desired aims: engaging the majority of Swiss acute care hospitals in a national project, achieving good representativeness of data and generating data that are comparable with other countries in Europe. Thus, although Switzerland did not formally take part in the second ECDC PPS in 2016 and 2017, applying the ECDC protocol allows benchmarking with EU/EEA countries.

## Conclusion

This was the first national PPS on antimicrobial consumption in Switzerland. Antimicrobial consumption is at European average, the use of broad-spectrum antibiotics in the lower third. Although national surveillance on AMR and antimicrobial consumption has been established, Swiss acute care hospitals should invest in antimicrobial stewardship, particularly in formalising responsibility and post-prescription review. Strategies to reduce antimicrobials, particularly broad-spectrum antibiotics, include the prevention of HAI and the reduction of prolonged surgical prophylaxis.

## Supplementary Data

123000 Supplement
