# Supplementary material for: Antimicrobial use in acute care hospitals: national point prevalence survey on healthcare-associated infections and antimicrobial use, Switzerland, 2017
Source: Euro Surveill. 2019 Aug 15;24(33):1900015. doi: 10.2807/1560-7917.ES.2019.24.33.1900015 (PMC6702796; doi:10.2807/1560-7917.ES.2019.24.33.1900015)
Supplement: Supplement [file 19-00015_ZINGG_Supplement.pdf]

Antimicrobial use in acute care hospitals: national point prevalence survey on healthcare-associated infections and antimicrobial use, Switzerland, 2017

Supplementary material

Disclaimer: "This supplementary material is hosted by *Eurosurveillance* as supporting information alongside the article [Antimicrobial use in acute care hospitals: national point prevalence survey on healthcare-associated infections and antimicrobial use, Switzerland, 2018] on behalf of the authors who remain responsible for the accuracy and appropriateness of the content. The same standards for ethics, copyright, attributions and permissions as for the article apply. *Eurosurveillance* is not responsible for the maintenance of any links or email addresses provided therein."

Supplementary Table 1. Distribution of indications for antimicrobial use, stratified by hospital size – Swiss national point prevalence survey on antimicrobial use 2017

Indication (4487 indications)	CH N; % (95%CI)	Hospital size			P-value
		<200 beds N; % (95%CI)	200-650 beds N; % (95%CI)	>650 beds N; % (95%CI)	
Therapy	(2808 indications)	(711 indications)	(995 indications)	(1102 indications)	0.002
Community-acquired infection	1886; 67.2 (65.4-68.9)	513; 72.2 (68.9-75.5)	726; 73.0 (70.0-75.7)	647; 58.7 (55.8-61.6)	<0.001
[1]Healthcare-associated infection	852; 30.3 (28.6-32.0)	173; 24.3 (21.1-27.5)	245; 24.6 (21.9-27.3)	434; 39.4 (36.5-42.3)	<0.001
LTCF-acquired infection	70; 2.5 (1.9-3.1)	25; 3.5 (2.2-4.9)	24; 2.4 (1.5-3.4)	21; 1.9 (1.1-2.7)	0.098
Prophylaxis	(1533 indications)	(481 indications)	(498 indications)	(554 indications)	<0.001
Medical prophylaxis	357; 23.3 (21.2-25.4)	44; 9.2 (6.6-11.7)	74; 14.9 (11.7-18.0)	239; 43.1 (39.0-47.3)	<0.001
Surgical prophylaxis	1176; 76.7 (74.6-78.8)	437; 90.9 (88.3-93.4)	424; 85.1 (82.0-88.3)	315; 56.9 (52.7-61.0)	<0.001
[2]*1 dose*	*677; 57.8 (54.7-60.4)*	*286; 65.5 (61.0-69.9)*	*261; 61.6 (56.9-66.2)*	*130; 41.3 (35.8-46.7)*	*<0.001*
[2]*1 day*	*149; 12.7 (10.8-14.6)*	*64; 14.7 (11.3-18.0)*	*59; 13.9 (10.6-17.2)*	*26; 8.3 (5.2-11.3)*	*0.022*
[2]*>1 day*	*350; 29.8 (27.1-32.4)*	*87; 19.9 (16.1-23.7)*	*104; 24.5 (20.4-28.6)*	*159; 50.5 (44.9-56.0)*	*<0.001*
Other or unknown indication	(146 indications)	(24 indications)	(52 indications)	(70 indications)	0.007

[1]Healthcare-associated as documented in the patient chart
[2]As per total surgical prophylaxis (1176 for CH; 437, 424, and 315 for small, medium-size, and large hospitals)
95%CI: 95%-confidence interval; LTCF: long-term-care facility

Supplementary Table 2. The ten most common antimicrobials – Swiss national point prevalence survey on antimicrobial use 2017

Rank	Antimicrobial (N = 5354)	N	Therapy									SP	MP	OTH
			All	CI	HI	BSI	LRTI	UTI	IA/GI	SSI/SST	Other			
1	CoAmox	1006 (18.7%)	765/1006 (76.0%)	579/765 (75.7%)	167/765 (21.8%)	56/765 (7.3%)	235/765 (30.7%)	50/765 (6.5%)	46/765 (6.0%)	306/765 (40.0%)	72/765 (9.4%)	170/1006 (16.9%)	40/1006 (4.0%)	31/1006 (3.1%)
2	Cefuroxime	612 (11.4%)	61/612 (10.0%)	51/61 (83.6%)	9/61 (14.8%)	3/61 (4.9%)	14/61 (23.0%)	11/61 (18.0%)	9/61 (14.8%)	21/61 (34.4%)	3/61 (4.9%)	528/612 (86.3%)	15/612 (2.5%)	8/612 (1.3%)
3	Ceftriaxone	542 (10.1%)	476/542 (87.8%)	391/476 (82.1%)	70/476 (17.7%)	52/476 (10.9%)	105/476 (22.1%)	145/476 (30.5%)	85/476 (17.9%)	33/476 (6.9%)	56/476 (11.8%)	38/542 (7.0%)	18/542 (3.3%)	10/542 (1.8%)
4	PipTaz	419 (7.8%)	382/419 (91.2%)	217/382 (56.8%)	161/382 (42.1%)	54/382 (14.1%)	136/382 (35.6%)	23/382 (6.0%)	81/382 (21.2%)	56/382 (14.7%)	32/382 (8.4%)	11/419 (2.6%)	9/419 (2.1%)	17/419 (4.1%)
5	Bactrim	309 (5.8%)	94/309 (30.4%)	54/94 (57.4%)	37/94 (39.4%)	6/94 (6.4%)	9/94 (9.6%)	59/94 (62.8%)	3/94 (3.2%)	12/94 (12.8%)	5/94 (5.3%)	36/309 (11.7%)	166/309 (53.7%)	13/309 (4.2%)
6	[1]Metronidazole	293 (5.5%)	209/293 (71.3%)	142/209 (67.9%)	61/209 (29.2%)	12/209 (5.7%)	11/209 (5.3%)	4/209 (1.9%)	142/209 (67.9%)	16/209 (7.7%)	24/209 (11.5%)	67/293 (22.9%)	8/293 (2.7%)	9/293 (3.1%)
7	Ciprofloxacine	265 (4.9%)	188/265 (70.9%)	117/188 (62.2%)	68/188 (36.2%)	14/188 (7.4%)	13/188 (6.9%)	70/188 (37.2%)	38/188 (20.2%)	38/188 (20.2%)	15/188 (8.0%)	59/265 (22.3%)	9/265 (3.4%)	9/265 (3.4%)
8	Cefazolin	226 (4.2%)	19/226 (8.4%)	10/19 (52.6%)	9/19 (47.4%)	4/19 (21.1%)	0/19 (0.0%)	0/19 (0.0%)	2/19 (10.5%)	8/19 (42.1%)	5/19 (26.3%)	205/226 (90.7%)	2/226 (0.9%)	0/226 (0.0%)
9	[2]Vancomycine	160 (3.0%)	150/160 (93.8%)	61/150 (40.7%)	87/150 (58.0%)	33/150 (22.0%)	7/150 (4.7%)	5/150 (3.3%)	18/150 (12.0%)	67/150 (44.7%)	20/150 (13.3%)	5/160 (3.1%)	0/160 (0.0%)	5/160 (3.1%)
10	Amoxicillin	129 (2.4%)	98/129 (76.0%)	76/98 (77.7%)	19/98 (19.4%)	16/98 (16.3%)	26/98 (26.5%)	10/98 (10.2%)	9/98 (9.2%)	20/98 (20.4%)	17/98 (17.3%)	17/129 (13.2%)	9/129 (7.0%)	5/129 (3.9%)

[1]Oral or parenteral
[2]Parenteral

Bactrim: Sulfamethoxazole and trimethoprime; BSI: Bloodstream infection (and sepsis); CI: Community-acquired infection; CoAmox: Amoxicillin and clavulanic acid; HI: Healthcare-associated infection; IA/GI: Intra-abdominal and gastrointestinal infection; LRTI: Lower respiratory tract infection; MP: Medical prophylaxis; OTH: Other indication for antimicrobial use; PipTaz: Piperacillin and enzyme inhibitor; SP: Surgical prophylaxis; SSI/SST: Surgical site-, soft tissue-, and bone and joint infection; UTI: Urinary tract infection

Supplementary Table 3. The ten most common combinations of two antimicrobials – Swiss national point prevalence survey on antimicrobial use 2017

Rank	Combinations of two antimicrobials (N = 638)	**N**	Therapy									SP	MP	OTH
			All	CI	HI	BSI	LRTI	UTI	IA/GI	SSI/SST	Other			
1	Ceftriaxone - Metronidazole	94 (14.7%)	72/94 (76.6%)	59/72 (81.9%)	11/72 (15.3%)	3/72 (4.2%)	3/72 (4.2%)	3/72 (4.2%)	53/72 (73.6%)	5/72 (6.9%)	5/72 (6.9%)	15/94 (16.0%)	5/94 (5.3%)	2/94 (2.1%)
2	Ciprofloxacine - Metronidazole	39 (6.1%)	32/39 (82.1%)	26/32 (81.3%)	6/32 (18.8%)	2/32 (6.3%)	0/32 (0.0%)	0/32 (0.0%)	25/32 (78.1%)	4/32 (12.5%)	1/32 (3.1%)	3/39 (7.7%)	1/39 (2.6%)	3/39 (7.7%)
3	Cefuroxime - Metronidazole	30 (4.7%)	9/30 (30.0%)	9/9 (100.0%)	0/9 (0.0%)	0/9 (0.0%)	0/9 (0.0%)	0/9 (0.0%)	8/9 (88.9%)	0/9 (0.0%)	1/9 (11.1%)	19/30 (63.3%)	0/30 (0.0%)	2/30 (6.7%)
4	Cefepime - Metronidazole	22 (3.4%)	22/22 (100.0%)	12/22 (54.5%)	10/22 (45.5%)	2/22 (9.1%)	6/22 (27.3%)	1/22 (4.5%)	4/22 (18.2%)	3/22 (13.6%)	6/22 (27.3%)	0/22 (0.0%)	0/22 (0.0%)	0/22 (0.0%)
5	Clindamycine - Ciprofloxacine	22 (3.4%)	20/22 (90.9%)	16/20 (80.0%)	4/20 (20.0%)	0/20 (0.0%)	3/20 (15.0%)	0/20 (0.0%)	1/20 (5.0%)	15/20 (75.0%)	1/20 (5.0%)	1/22 (4.5%)	1/22 (4.5%)	0/22 (0.0%)
6	CoAmox - Clarithromycine	22 (3.4%)	22/22 (100.0%)	18/22 (81.8%)	3/22 (13.6%)	1/22 (4.5%)	19/22 (86.4%)	1/22 (4.5%)	0/22 (0.0%)	0/22 (0.0%)	1/22 (4.5%)	0/22 (0.0%)	0/22 (0.0%)	0/22 (0.0%)
7	Ceftriaxone - Clarithromycine	20 (3.1%)	20/20 (100.0%)	20/20 (100.0%)	0/20 (0.0%)	0/20 (0.0%)	19/20 (95.0%)	0/20 (0.0%)	1/20 (5.0%)	0/20 (0.0%)	0/20 (0.0%)	0/20 (0.0%)	0/20 (0.0%)	0/20 (0.0%)
8	Meropeneme - Vanco	17 (2.7%)	15/17 (88.2%)	6/17 (40.0%)	9/17 (60.0%)	1/15 (6.7%)	2/15 (13.3%)	0/15 (0.0%)	3/15 (20.0%)	6/15 (40.0%)	3/15 (20.0%)	0/17 (0.0%)	0/17 (0.0%)	2/17 (11.8%)
9	PipTaz - Vanco	16 (2.5%)	16/16 (100.0%)	8/16 (50.0%)	8/16 (50.0%)	5/16 (31.3%)	1/16 (6.3%)	0/16 (0.0%)	3/16 (18.8%)	6/16 (37.5%)	1/16 (6.3%)	0/16 (0.0%)	0/16 (0.0%)	0/16 (0.0%)
10	Cefamandol - Metronidazole	14 (2.2%)	0/14 (0.0%)	0/14 (0.0%)	0/14 (0.0%)	0/0 (0.0%)	0/0 (0.0%)	0/0 (0.0%)	0/0 (0.0%)	0/0 (0.0%)	0/0 (0.0%)	14/14 (100.0%)	0/14 (0.0%)	0/14 (0.0%)

BSI: Bloodstream infection (and sepsis); CI: Community-acquired infection; HI: Healthcare-associated infection; IA/GI: Intra-abdominal and gastrointestinal infection; LRTI: Lower respiratory tract infection; MP: Medical prophylaxis; OTH: Other indication for antimicrobial use; PipTaz: Piperacillin and enzyme inhibitor; SP: Surgical prophylaxis; SSI/SST: Surgical site-, soft tissue-, and bone and joint infection; UTI: Urinary tract infection
